# Feasibility and preliminary effectiveness of the Individual Placement and Support (IPS) model for people with serious mental illness in Jalisco, Mexico: a pilot study protocol

**DOI:** 10.1186/s40814-026-01809-7

**Published:** 2026-05-01

**Authors:** Jesús Alejandro Aldana-López, Ricardo Saracco-Alvarez, Sol Durand-Arias, Jaime Carmona-Huerta, Elsy Cárdenas-García, Juan Carlos Arámbula-Román, Justin Metcalfe, Sarah Swanson, Jennie Keleher, Eveling Charrlot Villafuerte-Jacob, Denisse Flores-Bizarro, Jorge Antonio Blanco-Sierra, Robert E. Drake, Ezra Susser, Franco Mascayano

**Affiliations:** 1 Jalisco’s Institute of Mental Health, Zapopan, Mexico; 2 National Institute of Psychiatry, Mexico City, Mexico; 3 Westat, Rockville, MD USA; 4 IPS Employment Center, Research Foundation for Mental Hygiene, New York State Psychiatric Institute, New York, NY USA; 5 New York State Psychiatric Institute, New York, NY USA; 6 Dartmouth Psychiatric Research Center, Lebanon, NH USA; 7 Columbia University, New York, NY USA; 8 Department of Mental Health, Bloomberg School of Public Health, Johns Hopkins University, Baltimore, USA; 9 Global Mental Health Program, Institute of Public Health, Universidad Andrés Bello, Santiago, Chile; 10 Physiology Department. University Center for Health Sciences, University of Guadalajara, Guadalajara, Mexico; 11 Department of Medical Clinics, University Center of Tlajomulco, University of Guadalajara, Tlajomulco, Mexico

**Keywords:** Supported employment, Serious mental illness, Latin America, Individual placement and support, Global mental health

## Abstract

**Background:**

Severe mental illness (SMI) is associated with substantial barriers to competitive employment, including stigma, cognitive impairments, and limited social support. The Individual Placement and Support (IPS) model is an evidence-based intervention that provides individualized assistance for job search, placement, and retention. Although IPS has shown effectiveness in high-income countries, there is limited evidence regarding its feasibility and preliminary outcomes in low- and middle-income settings, particularly in Latin America.

**Methods:**

This pilot randomized controlled trial will assess the feasibility of implementing the IPS model for adults with SMI in Jalisco, Mexico. We will recruit 120 participants who are actively seeking employment and randomly assign them to either the IPS intervention or a control group receiving standard employment services. The intervention includes tailored support from trained IPS Employment Specialists in resume building, interview preparation, job search, and follow-up after job placement. Primary feasibility outcomes include recruitment and retention rates, participant acceptance, implementation fidelity, and identification of contextual barriers and facilitators. Secondary outcomes will explore employment status at 12 months, financial well-being, and health-related outcomes.

**Discussion:**

Findings from this pilot study will contribute to addressing the current gap in implementation research on IPS in Latin America. Findings will offer preliminary insights into the feasibility, acceptability, and contextual adaptability of the model in a public mental health setting. These results are expected to guide the refinement of study procedures and support planning for a future definitive trial. Additionally, exploratory data on employment and quality of life outcomes may help identify relevant domains for further investigation.

**Trial registration:**

ClinicalTrials.gov NCT06019247. Registered on August 31, 2023.

## Introduction

### Background and rationale

Severe mental illness (SMI) limits access to competitive employment due to persistent stigma, cognitive challenges, and reduced social support. Employment has been associated with improved quality of life, self-esteem, and social integration among individuals with SMI, as well as reductions in symptom severity, hospitalization rates, and mental health service costs [1]. The Individual Placement and Support (IPS) model is a structured, evidence-based approach to supported employment that offers individualized assistance for job search, placement, and retention. Its core principles and operational components are outlined in Table 1 [2].

**Table 1:** Principles and practices of the Individual Placement and Support (IPS) model

Principles	Practices
Competitive Employment: Agencies providing IPS are committed to regular jobs in the community as an attainable goal for clients seeking employment	Job Search and Placement: IPS Employment specialists assist clients in identifying job opportunities, preparing resumes, practicing interview skills, and applying for jobs
Zero Exclusion: Every client who wants to work is eligible for services regardless of “readiness”, work experience, symptoms, or any other issue	Initial Assessment and Vocational Profile: An initial assessment gathers information about the client’s work history, skills, interests, and preferences to guide the job search and placement process
Attention to Clients’ Preferences: Services align with clients’ choices, rather than practitioners’ expertise or judgments; IPS Employment Specialist help clients find jobs that fit their preferences and skills	Worker Preferences: Services are based on each job seeker’s preferences and choices, ensuring alignment with the individual's career goals and personal interests
Rapid Job Search: IPS programs help a client look for jobs soon after he/she expresses interest in working, rather than providing lengthy pre-employment assessment, training, and counseling	Immediate Job Search Initiation: Job searches begin within 30 days of program entry to minimize delays and ensure timely job application processes
Targeted Job Development: Based on clients’ interests, IPS Employment Specialist build relationships with employers through repeated contact, learning about the business needs of employers, and introducing employers to qualified job seekers	Systematic Job Development: IPS Employment Specialist visit employers to learn about their business needs and match them with clients' interests and skills
Integration of Employment Services with Mental Health Treatment: IPS programs closely integrate with mental health treatment teams	Integrated Services: IPS Employment specialists work as part of mental health treatment teams to provide coordinated care and comprehensive support
Personalized Benefits Counseling: IPS Employment Specialist help clients obtain personalized, understandable, and accurate information about how working may impact their disability insurance and other government entitlements	Benefits Planning: IPS Employment Specialist provide accurate information about Social Security, Medicaid, and other entitlements to help clients make informed decisions
Individualized Long-Term Support: Follow-along supports, tailored for the individual, continue for as long as the client wants and needs them to keep a job or advance career opportunities	On-the-Job and Follow-Along Support: Continuous, individualized support is provided to help clients retain employment and advance in their careers

Studies in high-income countries consistently report higher employment rates among IPS participants, typically around 55%, with some trials showing rates above 60% compared to standard vocational services [3]. However, there is limited evidence on IPS implementation and outcomes in low- and middle-income countries (LMICs), especially in Latin America. Regional studies highlight structural disadvantages among individuals with SMI in this region, including poverty, unemployment, interrupted education, and exposure to trauma and violence [4–6].

In Mexico, mental health care for people with SMI is often fragmented and misaligned with evidence-based practices [7]. The Jalisco public mental health system is a notable exception. It includes the Center for Comprehensive Care in Long-Stay Mental Health, the state’s largest psychiatric facility, which provides primary care, pharmacological treatment, and psychosocial rehabilitation [8]. However, existing employment services are generally lower in intensity and do not incorporate key IPS components, resembling services offered in other LMIC urban settings [9].

Recent reforms in Jalisco offer a favorable context for implementing and testing IPS. First, state legislation now guarantees the right to community-based mental health care, including free public access for individuals with SMI [10]. Second, a network of community-based services has expanded to include employment-oriented psychosocial rehabilitation activities [8]. Third, the National Employment Service supports a labor inclusion strategy targeting job seekers with psychosocial disabilities, in line with anti-discrimination laws and labor policy frameworks [11].

We propose a pilot study to explore context-informed adaptations of the IPS model and its implementation for people with SMI in Jalisco, Mexico, using an explanatory sequential mixed-methods design embedded within a pilot randomized controlled trial. Quantitative and qualitative data will be collected across phases to assess feasibility, fidelity, and contextual implementation barriers [12]. Findings from this pilot will contribute to the design of a future definitive trial and offer preliminary insights that could inform future research on the adaptation of employment support services in Mexico and other LMICs.

### Aims and objectives

The primary aim of this pilot randomized controlled trial is to evaluate the feasibility and contextual fit of an adapted version of the Individual Placement and Support (IPS) model for people with serious mental illness (SMI) within the Jalisco mental health system in Mexico. The specific objectives are:

1. To adapt the IPS model to the Jalisco context, based on input from stakeholders including policymakers, service providers, users, and employers. This process will identify contextual barriers and inform modifications to IPS materials, delivery format, and fidelity criteria. The adapted model will be implemented through blended training (virtual and in-person sessions, e-learning resources).
2. To assess the feasibility of implementing the adapted IPS model, including recruitment and retention rates, acceptability among stakeholders, and fidelity of delivery. Qualitative data will be collected to explore perceived facilitators and barriers to implementation.
3. To explore preliminary outcomes related to employment, financial well-being, and health among participants. Outcomes will be compared between intervention and control groups at baseline, 6 months, and 12 months.
4. To evaluate implementation processes and resource needs, including patterns of participation and fidelity over time, multilevel barriers and facilitators (e.g., at the provider and system level), and resource requirements (e.g., personnel, time, and infrastructure) for potential scale-up.

### Trial design

This mixed-method pilot randomized controlled trial (RCT) will evaluate the feasibility and contextual fit of an adapted IPS model for individuals with SMI in the Jalisco mental health system. We apply the Dynamic Adaptation Process (DAP) model [13], an implementation science framework that allows for structured, iterative adaptations to evidence-based practices while maintaining fidelity to their core components. The four phases of the study—Preparation, Adaptation, Implementation, and Evaluation—reflect the DAP structure and inform the sequencing of study activities to guide IPS adaptation and implementation, assess fidelity, and identify contextual barriers and facilitators.

We will evaluate the implementation and preliminary outcomes of the adapted intervention through a pilot randomized controlled trial (*N* = 120). This study includes quantitative and qualitative assessments at baseline, 6, and 12 months, involving participants (e.g., employment, financial well-being), providers (e.g., attitudes towards evidence-based practices), and other key stakeholders (e.g., potential employers). This design addresses the primary aim and specific objectives described above by allowing us to examine implementation processes, explore patterns of employment and health-related outcomes, and generate estimates needed to plan a future regional trial in Latin America.

Prior experiences with IPS adaptations in other countries and marginalized populations in the USA (e.g., Latinxs, individuals experiencing homelessness) [14, 15]—together with insights from the local Mexican labor market—inform our adaptation strategy. We completed initial interviews and focus groups with stakeholders as described below.

**Fig. 1:**
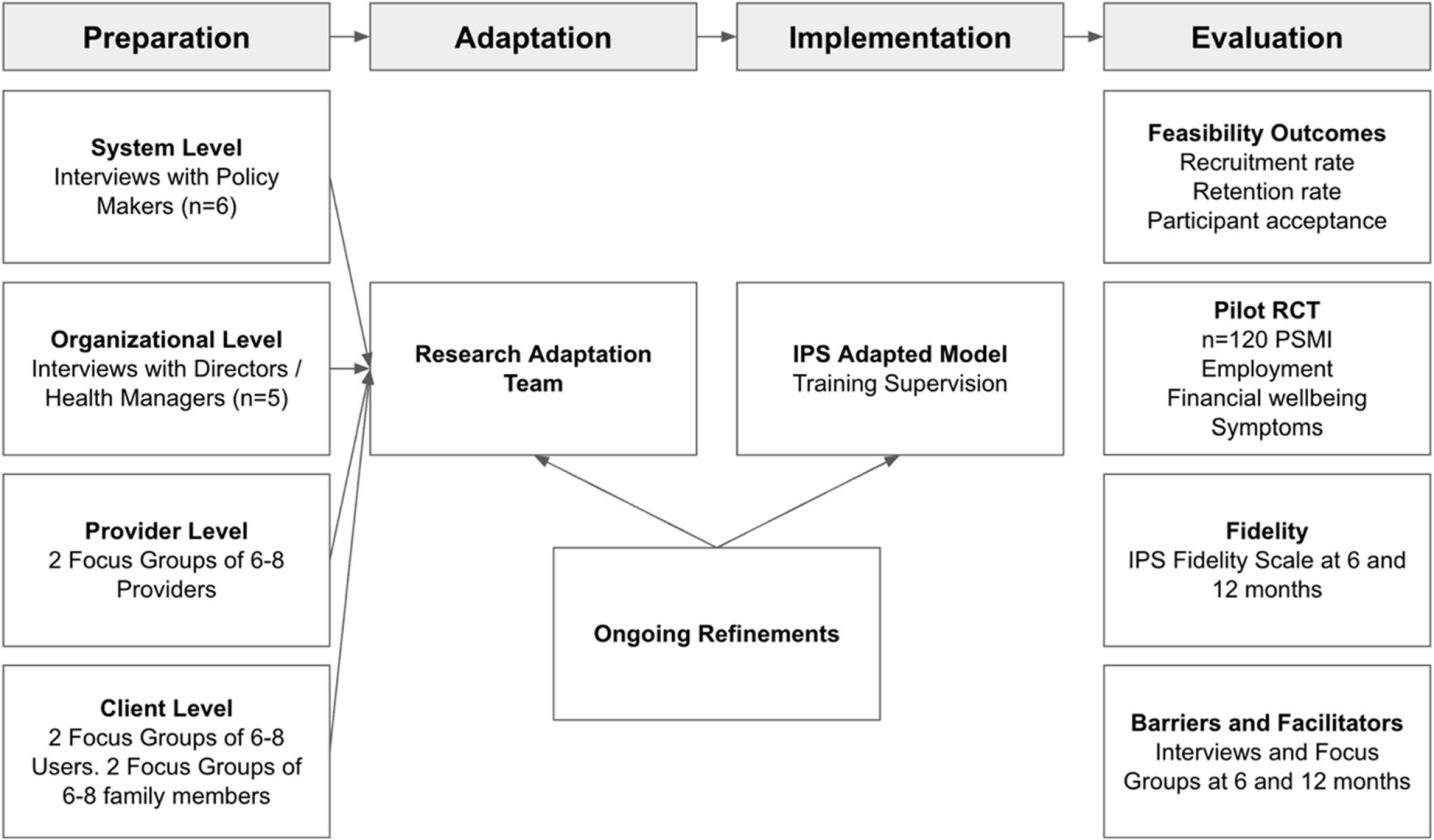
Phased implementation strategy for the adapted IPS model guided by the Dynamic Adaptation Process (DAP). The diagram illustrates the study’s four phases—Preparation, Adaptation, Implementation, and Evaluation—each with its corresponding activities. The Evaluation phase includes assessment of feasibility outcomes (recruitment, retention, and participant acceptance), as well as implementation fidelity, contextual barriers, and pilot outcome measures. Stakeholder engagement at all levels informs adaptations and supports continuous refinement of the IPS model

IPS implementation follows four DAP phases: preparation, adaptation, implementation, and evaluation (Fig. 1). Collaborators in the USA have used a similar approach to deliver other evidence-based programs in Latin America, such as OnTrack Chile (1U01MH115502-01) [16]. This iterative process integrates ongoing experience to refine adaptations. We engage multiple stakeholders (e.g., policymakers, providers, participants, and employers) to discuss contextual factors within and beyond mental health services and identify potential adaptations to the IPS curriculum. Once the trial begins, ongoing training will involve in-person meetings and e-learning modules. We will apply mixed-methods to assess fidelity, service delivery patterns, contextual barriers and facilitators, and estimated implementation costs. The specific objectives, outcomes, methods, and expected outputs for each phase are summarized in Table 2.

**Table 2:** Study phases, objectives, implementation outcomes, methods, and expected outputs

Phase	Objective	Implementation outcome(s)	Methodology	Expected outputs
Preparation	Understand contextual barriers and assess readiness	Organizational readiness	Semi-structured interviews, focus groups	Matrix of barriers and facilitators
Adaptation	Tailor IPS model while preserving fidelity to core components	Contextual fit, fidelity	Stakeholder meetings, material review, iterative feedback	Adapted IPS materials; training and supervision plan
Implementation	Deliver IPS model with fidelity in routine services	Fidelity, acceptability	IPS service delivery; fidelity assessment; training logs	Fidelity reports, training logs, implementation notes
Evaluation	Assess feasibility and inform the design of a future trial	Feasibility, retention, acceptance	Pilot RCT; follow-up interviews and focus groups	Descriptive statistics; thematic analysis reports

## Methods: participants, interventions, and outcomes

### Study setting

The study takes place in the Jalisco public mental health system, as previously described [8], which integrates community-based services and offers a structural foundation for implementing and testing the IPS model in a real-world context. This setting engages multiple stakeholders, including policymakers, healthcare providers, service users, employers, and the National Employment Service. These actors contribute to the adaptation and delivery of the IPS model, aligning it with local needs and service structures.

The National Employment Service brings expertise in employment access for people with disabilities, including those with mental health conditions [10], and supports participant job placement efforts. The setting provides the institutional infrastructure and community reach required to assess the feasibility of the IPS model in a Latin American public health system. Findings may inform future implementation planning in similar contexts.

### Eligibility criteria

Participants are eligible if they are at least 18 years old and have a diagnosis consistent with SMI, including schizophrenia spectrum disorders, bipolar I disorder, major depressive disorder with severe functional impairment, or other psychotic disorders. Diagnoses will be made by the treating psychiatrist at the Center for Comprehensive Care in Long-Stay Mental Health as part of routine clinical evaluation. No symptom severity cut-offs will be used for eligibility; instead, SMI will be determined based on the presence of a persistent psychiatric disorder associated with substantial functional impairment in daily activities. This approach is consistent with international definitions of SMI and with current clinical practice at the study site. Additional eligibility criteria include expressing a goal of obtaining paid, competitive employment, attending two informational sessions prior to recruitment, having no competitive employment in the past 3 months, being available to participate in the study for 1 year of follow-up, and providing informed consent.

### Interventions

#### Explanation for the choice of comparators

Treatment as usual (TAU) for individuals with SMI at the Center for Comprehensive Care in Long-Stay Mental Health is delivered by a multidisciplinary team composed of two full-time psychologists, two full-time social workers, a part-time occupational therapist, and a part-time psychiatrist. This team maintains connections with various public and private organizations that assist in job placement for individuals with mental disabilities, including cooperatives. The positions offered through these channels are generally categorized as non-competitive or self-employment activities, such as broom-making, craft-making, or gardening.

In addition to vocational support, the treatment team provides psychiatric medication, individual and group psychotherapy, and psychoeducation for both clients and their families. They also serve individuals with other mental health conditions, including substance use disorders. Vocational services offered in this setting are less intensive than those delivered through the IPS model and do not incorporate its core components. Furthermore, usual care is not guided by a standardized manual, protocol, or fidelity framework.

Usual care (TAU) was selected as the comparator because it represents the standard level of vocational and mental health services currently available in the Jalisco public mental health system. TAU is the ethically appropriate comparator given that no evidence-based supported employment model is routinely implemented in this setting. Using TAU allows evaluation of the added value of IPS within a real-world system and aligns with recommendations for pilot and feasibility trials of IPS in low- and middle-income countries, where comparisons with existing services enhance external validity. Furthermore, prior IPS trials in Latin America and other LMIC regions have relied on TAU as the primary comparator, making this choice consistent with international methodological practice.

#### Intervention description

The adaptation of the IPS model to the study sites has been described previously. Any changes indicated through the Dynamic Adaptation Process might result in unanticipated modifications to the IPS model. However, it is expected that the adaptation process will not compromise the core principles of the IPS model. A trained IPS technical assistance/quality-assurance expert will oversee this process, providing ongoing IPS implementation support and facilitating consistent communication among clinicians, researchers, and the IPS team.

Following randomization, participants allocated to the intervention group will be referred to the IPS team based at the National Employment Service office. This team consists of one IPS Supervisor and two IPS Employment Specialists, all current staff members of the Employment Service. They have completed initial training and will participate in weekly meetings to reinforce their knowledge of the IPS model. Each IPS Employment Specialist will manage a caseload of approximately 15 to 20 participants.

The IPS team will participate in weekly vocational unit meetings, integrated treatment team meetings with clinical staff from the Center for Comprehensive Care in Long-Stay Mental Health, and regular individual supervision sessions. In accordance with the IPS model, the IPS Employment Specialists will develop relationships with employers based on participants’ work preferences, focusing on competitive employment opportunities open to individuals with and without disabilities. They will also coordinate follow-along support to help participants sustain employment. To support the implementation process, an experienced IPS trainer (JK) will join vocational unit meetings weekly and clinical team meetings monthly via video conference. The trainer will also offer remote consultation by phone or online, as needed, throughout the implementation phase.

#### Criteria for discontinuing or modifying allocated interventions

Participation in the study is voluntary, and participants may withdraw at any time without penalty. The intervention may also be temporarily paused or discontinued if the clinical team determines that continued participation is not advisable due to acute deterioration or circumstances that interfere with study procedures. These decisions will be made in coordination with the participant’s usual clinical providers.

Although measures are in place to safeguard data privacy and confidentiality, there remains a potential risk of unauthorized disclosure. To minimize this risk, the study will adhere to strict data protection protocols and ensure that research interviewers remain blinded to group assignment.

#### Risk and risk management

Participants will be adults with SMI receiving community-based services; therefore, some level of clinical and psychosocial risk is expected. A structured risk management protocol will be implemented to ensure participant safety throughout the trial. All IPS Employment Specialists and research staff will be trained to identify warning signs related to clinical deterioration, including increased symptoms, functional decline, suicidal ideation, or psychosocial crises.

If a participant reports suicidal thoughts, expresses intent, or presents significant behavioral changes suggesting elevated risk, the IPS Employment Specialist will pause all study-related activities and immediately activate the clinical escalation protocol. This protocol includes: (1) notifying the participant’s treating psychiatrist or mental health provider; (2) conducting a brief safety check using established procedures from local mental health services; and (3) coordinating a same-day clinical evaluation when indicated. In emergency situations, specialists will contact emergency services according to institutional guidelines.

Study participation may be temporarily paused or discontinued if the participant experiences acute psychiatric decompensation, requires hospitalization, or if the clinical team determines that continued involvement poses an unacceptable risk. All risk events and procedures will be documented and reviewed by the Research Adaptation Team and the site’s clinical supervisors to ensure consistent application of risk management procedures. The protocol prioritizes continuity of clinical care, and all participants will remain within their routine mental health services regardless of trial involvement.

#### Strategies to improve adherence to interventions

High adherence to the intervention is expected due to the structured nature of the IPS model, which allows for contextual and individual-level adaptations. Adherence will be monitored by IPS experts from the USA and the research team in Mexico, who will collaborate to ensure fidelity to the model while tailoring implementation to the local context in Jalisco. One of the IPS trainers is Spanish-speaking, facilitating direct support to local staff. To monitor compliance, the study will utilize structured tools, including activity logs, monthly implementation reports, and periodic assessments using an IPS fidelity scale.

#### Relevant concomitant care permitted or prohibited during the trial

Participants in both study arms will continue to receive standard mental health services at the Center for Comprehensive Care in Long-Stay Mental Health. These services are delivered by a multidisciplinary team including psychiatrists, psychologists, social workers, occupational therapists, and nurses. Standard care includes psychiatric medication, individual psychotherapy, and psychoeducation primarily focused on clients and family members. It does not incorporate the individualized, recovery-oriented components characteristic of the IPS model.

#### Provisions for post-trial care

Participants will continue receiving their usual clinical care at the Center for Comprehensive Care in Long-Stay Mental Health after the study concludes. No study-specific treatments will be withdrawn at the end of the trial, and no additional risks are expected following participation. As this is a minimal-risk study, no post-trial compensation or ancillary care is required. Participants will remain connected to their regular clinical teams, who will provide ongoing care as clinically indicated.

#### Outcomes

This study will assess feasibility outcomes related to the implementation of the IPS model and explore its preliminary effects on participant employment, financial well-being, and health-related outcomes. The evaluation will emphasize feasibility outcomes, while participant-centered outcomes will provide additional insight into the model’s potential impact. Data will be collected at baseline and through quarterly follow-up interviews. All instruments will be administered in Spanish; if no validated Spanish version exists, the measure will be translated using forward–back translation by bilingual specialists, followed by expert panel review to ensure conceptual equivalence and linguistic clarity. Table 3 provides a detailed summary of all planned outcomes and associated measures.

**Table 3:** Outcomes and measures

Measure	Data Source	Interval (duration)	Constructs Addressed
Baseline participant interview	Participant	Baseline (45–60 min)	- Demographics (age, sex, gender, marriage status, race/ethnicity)—employment history (past year and past 5 years)—income history (annual income, income sources)—Housing history (past 5 years)—health service utilization history (past-year hospitalization and emergency service utilization, regular medical care)—health and functionality (Brief Psychiatric Rating Scale (BPRS), Medical Outcomes Study Short-Form 12-Item (SF-12, mental and physical), Social Occupational Functioning Scale (SOFS), Recovery Assessment Scale (RAS-24)—Social capital (numbers, education, and justice system involvement of parents, siblings, children, and friends)—Justice system involvement (arrest, incarceration, and conviction history)
Quarterly employment interview	Participant	Quarterly (10 min)	- Past-quarter employment (any competitive employment; per-job weeks worked, hours worked per week, hourly wage, job start/end dates if applicable)
Follow-up participant interview	Participant	6, 12 months (40–50 min)	- Housing (past 6 months)—health service utilization (past 6 months hospitalization and emergency service utilization)—health and functionality (Brief Psychiatric Rating Scale (BPRS), Medical Outcomes Study Short-Form 12-Item (SF-12, mental and physical), Social Occupational Functioning Scale (SOFS), Recovery Assessment Scale (RAS-24)
IPS Participation Measure	IPS Employment Specialist	Monthly (5–10 min)	- Observed participant: past-month competitive employment status (employed/not employed)—past month participation in IPS services (yes/no)—IPS activities participated in—past-month contact with IPS team (yes/no)—reasons for being either out of contact or not participating in IPS services—qualitative: difficulties encountered engaging participant or working with employers
Employment Specialist Service Log	IPS Employment Specialist	Monthly (5–10 min)	- IPS Employment Specialist past-week time spent in: direct service (vs. indirect)—the community (vs. in the office)—Collaborating with mental health clinicians in the clinic—Job development—Job support

#### Primary feasibility outcomes

- Recruitment and retention rates: the study will monitor the number of participants enrolled and retained throughout the trial to assess whether the target sample size can be achieved within the planned timeframe.
- Fidelity of IPS implementation: adherence to core IPS principles will be measured using the IPS Fidelity Scale.
- Acceptability and perceived barriers: qualitative interviews and focus groups with stakeholders (e.g., participants, providers, employers) will explore the acceptability of the IPS model and perceived barriers to its implementation.

#### Secondary participant-centered outcomes

These outcomes will capture individual-level changes related to employment, financial stability, and health. A detailed schedule of assessments is presented in Table 3.

- Employment outcomes: monthly employment status (employed/not employed), job start and end dates, average hours worked per week, and hourly wages.
- Financial well-being: annual income, income sources, and housing stability over the past 12 months.
- Health-related outcomes: use of health services (e.g., hospitalizations, emergency visits) and self-reported measures of general health and functioning.

#### Progression criteria for a future definitive trial

Progression to a future definitive trial will be guided by a pre-specified traffic-light framework across four feasibility domains: recruitment, retention, IPS fidelity, and acceptability. Transparent “red–amber–green” thresholds will guide decisions regarding continuation, amendment, or discontinuation of a future trial (Table 4).

- Recruitment will be assessed as the proportion of achieved versus planned recruitment at matched timepoints during the recruitment period. Recruitment performance will be classified as green if ≥ 85% of the planned recruitment target is achieved, amber if 70–84% is achieved, and red if < 70% is achieved.
- Retention will be evaluated at the primary follow-up timepoint (12 months). Retention will be classified as green if ≥ 77% of participants are retained, consistent with the retention benchmark anticipated in the study protocol, amber if retention is 60–76%, and red if retention is < 60%.
- IPS fidelity will be assessed using the IPS-25 Fidelity Scale, a validated instrument widely used to evaluate adherence to the IPS model. Fidelity will be classified as green if the program achieves a score ≥ 100 (indicating good fidelity), amber if scores fall between 74 and 99 (fair fidelity requiring improvement), and red if scores are ≤ 73 (not IPS fidelity).
- Acceptability will be evaluated using a combination of quantitative participant ratings and qualitative interview data. Quantitative acceptability ratings will be summarized descriptively, and qualitative findings will be interpreted within established implementation science frameworks. Acceptability will be classified as green when quantitative ratings indicate generally favorable perceptions and qualitative data reveal no major barriers to participation; amber when mixed perceptions or remediable barriers are identified; and red when qualitative evidence identifies substantial concerns or barriers that would likely preclude successful implementation of a definitive trial.

**Table 4:** Pre-specified progression criteria for feasibility outcomes

Feasibility domain	Green (proceed)	Amber (amend)	Red (stop/reconsider)
Recruitment	≥ 85% of target recruitment achieved	70–84% achieved	< 70% achieved
Retention (12 months)	≥ 77% retained	60–76% retained	< 60% retained
IPS fidelity (IPS-25 scale)	≥ 100 (good fidelity)	74–99 (fair fidelity)	≤ 73 (not IPS fidelity)
Acceptability	Favorable participant ratings and no major implementation barriers	Mixed perceptions or remediable barriers	Major concerns or barriers likely to prevent successful implementation

Progression to a definitive trial will be recommended when no more than one domain is rated red and the overall pattern of findings indicates that identified barriers are remediable and do not threaten the feasibility of the study procedures. In such cases, a remediation plan addressing the red domain will be implemented within a predefined timeframe (≤ 60 days). Amber ratings will trigger predefined amendments to study procedures or implementation strategies. If two or more domains are rated red, the study will be considered not feasible to proceed without substantial redesign.

#### Participant timeline

The schedule of enrollment, intervention delivery, and outcome assessments is summarized in Table 5. Participants will be followed for 12 months, with assessments conducted at baseline and quarterly thereafter.

**Table 5:** Participant timeline

Study period	Enrollment	Allocation	Post-allocation	Close-out
Timepoint (quarterly)	-q1	0	q1	q2
Enrollment:				
Eligibility screen	X			
Informed consent	X			
Allocation		X		
Interventions:				
IPS services			X	
Usual services			X	
Assessments:				
Baseline variables	X	X		
Quarterly employment interview			X	X
Follow-up participant interview			X	
IPS Participation Measure (monthly)			X	X
IPS Employment Specialist Log (monthly)			X	X

#### Sample size

The study will include a convenience sample of 120 participants, with 60 allocated to each group. As this is a pilot feasibility trial, no formal power calculation was conducted. Instead, the sample size was determined based on feasibility considerations, alignment with previous IPS pilot studies in similar contexts [17, 18], and the need to generate stable estimates of recruitment, retention, and preliminary variability in outcomes.

This approach is consistent with current methodological guidance for feasibility studies, which emphasizes that sample sizes should be justified according to their ability to assess feasibility parameters rather than to detect statistically significant effects. Feasibility trials aim to inform the design and sample size of a future definitive randomized controlled trial rather than to conduct hypothesis testing or achieve statistical power. The selected sample size is therefore appropriate to evaluate the acceptability, feasibility, and preliminary implementation of the adapted IPS model in this setting and to inform the parameters required for a fully powered RCT [19].

#### Recruitment

The study will implement several recruitment strategies over a 12-month period to enroll 120 participants with 1 year of planned follow-up. Clinicians at the Center for Comprehensive Care in Long-Stay Mental Health will identify potential participants during routine care and refer them to informational sessions offered by the psychosocial rehabilitation service. Promotional materials (e.g., posters and flyers) will be placed in waiting areas and hallways to raise awareness.

Research staff will conduct informational sessions and provide individual outreach to support ongoing recruitment. Individuals who attend a session and express interest in employment will be screened for eligibility, complete the informed consent process, undergo randomization to either usual care or the adapted IPS intervention, and complete the baseline assessment.

Recruitment projections are based on previous IPS trials that achieved consistent enrollment through sustained collaboration with clinical teams and use of incremental recruitment targets [20]. Participant feedback and service utilization data will be used to refine recruitment procedures throughout the study.

### Assignment of interventions: allocation

#### Sequence generation

This study follows a randomized, controlled, outcome-assessor-blinded, parallel-group design. To ensure balance between the intervention (IPS) and control (Usual Care) groups, permuted block randomization with a block size of four will be used. Accounting for an expected 25% attrition rate, the study will recruit 160 participants, arranged in 40 blocks, to meet the target sample size of 120 participants.

#### Concealment mechanism

A study statistician will generate the randomization sequence in advance using permuted blocks. The sequence will be stored securely in the REDCap system and will not be accessible to study staff, except for the statistician. After the baseline interview, REDCap will assign the intervention based on the pre-generated sequence. A research assistant will facilitate this allocation process during the second recruitment interview.

#### Assignment of interventions: blinding

Due to the nature of the intervention, it is not feasible to blind participants, clinical staff delivering IPS, or the research team involved in its implementation. However, interviewers conducting baseline and follow-up quantitative assessments—covering employment, hospitalization, symptoms, functioning, and quality of life—will remain blinded to group assignment. Data analysts and statisticians will also be blinded during the analysis phase.

#### Procedure for unblinding if needed

As this is an open-label study, no standard procedure for unblinding is required during routine operations. However, if a serious adverse event or exceptional circumstance arises that necessitates revealing a participant’s group assignment, the principal investigator may access the randomization record. Any instance of unblinding, along with its rationale, will be documented and reviewed to ensure adherence to study protocols and ethical standards.

### Data collection and management

#### Plans for assessment and collection of outcomes

Baseline structured interviews will be conducted prior to randomization to collect data on demographic characteristics, social capital, physical and mental health status, health service utilization, justice system involvement, functionality, and employment and financial history [21]. Validated instruments with established psychometric properties and Spanish versions will be used at baseline and follow-up assessments. These include the Brief Psychiatric Rating Scale (BPRS) for psychiatric symptoms [22], the Medical Outcomes Study Short-Form 12-Item (SF-12) for physical and mental health [23], the Social and Occupational Functioning Scale (SOFS) for functioning and role performance [24], and the Recovery Assessment Scale (RAS-24) to assess perceived recovery and empowerment [25]. All instruments will be administered by trained research staff using structured protocols to ensure consistency and data quality.

Quarterly structured follow-up interviews will assess employment status during the prior 3 months. Data collected will include job titles, employment dates, hours worked, and wages earned. When applicable, the reason and timing of job termination will also be recorded [21]. These structured interviews will be conducted in person at the Center for Comprehensive Care in Long-Stay Mental Health, subject to public health restrictions. If a participant is unavailable, the research team will make multiple contact attempts within the same data collection window. Missed interviews will be documented, and retrospective data will be gathered at the next opportunity.

Follow-up structured research interviews scheduled at 6- and 12 months after randomization, the follow-up interviews will augment the quarterly employment interviews and include measures of health and functionality mirroring those administered at baseline. Additional data collected describes housing, social capital, and health service utilization [21]. Follow-up interviews will be conducted at the same time as the corresponding quarterly employment interviews. As with the quarterly employment interviews, when participants are unavailable for follow-up interview, the research assistant will make several efforts to track and schedule the interview within the designated time until the next quarterly interview period.

IPS fidelity will be assessed using the 25-item IPS Fidelity Scale (IPS-25), a validated instrument with strong internal consistency and predictive validity [26]. Fidelity assessments will take place at approximately 6 and 12 months after program initiation. Experienced IPS reviewers will conduct in-person evaluations, including interviews with IPS staff, clinical team members, administrators, clients, and family members. Observations will cover vocational unit meetings, team discussions, and employer engagements. Reviewers will also examine selected participant records. Results will be used to monitor fidelity, highlight areas for adaptation, and guide ongoing quality improvement efforts.

The IPS Participation Measure is a brief form completed monthly by IPS Employment Specialists to track client engagement and services delivered (e.g., job search, career profiling, support provision) [21]. This tool captures information on employment status, IPS service participation, and reasons for non-participation [27]. IPS Employment Specialists will complete and submit the form monthly for each participant assigned to them. This measure was used with high completion rates in the Supported Employment Demonstration project and allows for the monitoring of IPS adaptations over time [28]. Tracking participation status and reasons for disengagement will support ongoing efforts to improve participant engagement in the IPS program.

Each month, IPS Employment Specialists will complete a service log documenting IPS activities performed during the previous week. This time-sampled approach has shown greater reliability than continuous measurement methods. Logs will capture time spent on various service categories, including direct versus indirect services, community-based versus in-office activities, collaboration with clinic-based mental health staff, job development (e.g., in-person employer meetings), and the delivery of job support. These data will allow for comparisons with current IPS program standards associated with high fidelity. Additionally, the information will help assess the extent to which IPS service delivery has been adapted through the Dynamic Adaptation Process.

External interviewers with experience in clinical and social assessments will be recruited and trained prior to data collection. Both interviewers and IPS employment specialists will enter data using standardized online forms stored on a secure server. Centralized data entry will enable the study analyst to monitor data quality, identify missing or incorrect entries, and adjust data collection procedures as needed to ensure data integrity. All data collection activities will comply with the confidentiality protocols of the Center for Comprehensive Care in Long-Stay Mental Health.

Each quarter, the IPS Supervisor will receive a summary report based on the IPS employment specialists’ service logs. These summaries will support monitoring of IPS implementation and help identify areas requiring adjustment in collaboration with the IPS central team. The IPS Trainer will also receive the reports to assist the Supervisor in addressing challenges and reinforcing adherence to core IPS principles.

#### Plans to promote participant retention and complete follow-up

This study enrolls clients who are already receiving services at the participating site and have established relationships with clinical providers. Combined with pre-recruitment informational sessions and the ongoing engagement of clinicians and research staff, this approach has yielded high retention rates—exceeding 90% at 1- and 2-year follow-up—in similar studies. However, given the limited precedent for IPS implementation in Mexico, we account for a more conservative retention estimate. For example, the Supported Employment Demonstration reported a 77% 1-year IPS participation rate [28]. Accordingly, we anticipate a retention rate of approximately 77%, and this figure informed our sample size and recruitment planning to ensure adequate follow-up data.

To maximize retention and minimize attrition, we will implement several strategies: clearly explaining study requirements prior to enrollment; conducting assertive outreach; collecting updated locator information at each contact point; maintaining regular communication; and providing compensation for each completed interview. Participants will remain eligible for follow-up assessments regardless of their level of engagement with IPS services. Prior studies suggest that participants often stay engaged due to a desire to share their experiences and contribute to the improvement of mental health services.

## Data management

All trial data will be entered into a REDCap database [29] hosted by the research site. The system will track the completion of all study forms, including baseline, quarterly, and final assessments, as well as IPS participation data. Access will be password-protected, and users will only be able to access their assigned records. Any data entry errors will be corrected by authorized research staff, who will document the changes and the reasons for each correction. REDCap automatically maintains an audit trail of all edits and user activity. The Principal Investigator (PI) or designee will implement a data verification protocol and generate reports at the end of enrollment to monitor recruitment progress and assess covariate balance across study arms. The study statistician will review data completeness and IPS participation quarterly. At the end of the trial, data will be exported from REDCap as self-documenting SAS files for cleaning and analysis.

### Statistical methods for primary and secondary outcomes

- Primary feasibility outcomes will be analyzed descriptively. Recruitment and retention will be summarized as proportions with 95% confidence intervals. Fidelity scores will be summarized using total and domain scores at each assessment point. Acceptability findings will be integrated descriptively with qualitative data. Quantitative analyses will follow an intent-to-treat approach. Employment, financial well-being, and health-related outcomes will be analyzed using linear generalized mixed models, accounting for clustering at the site level. Effect sizes will be reported with 95% confidence intervals to assess the direction and magnitude of potential impacts. Preliminary analyses will examine data distributions and apply transformations where necessary. Baseline characteristics will be summarized descriptively by randomized group to describe the study sample. No statistical tests will be conducted to compare baseline variables between groups, as any differences are expected to occur by chance due to randomization. Where appropriate, pre-specified prognostic covariates may be included in adjusted models to improve precision of effect estimates. Secondary analyses will include employment trajectories, non-vocational outcomes, and analyses based on level of exposure to the intervention. Longitudinal data will be analyzed using mixed-effects models. Time-to-event outcomes will be evaluated using Kaplan–Meier curves and Cox proportional hazards models.
- Qualitative data will be analyzed using a flexible coding approach. An initial multilevel codebook (systemic, organizational, provider, participant) will guide the deductive phase of coding, followed by the identification of inductive codes emerging from transcript review. Coding will be conducted by multiple team members who will meet regularly to refine the codebook and resolve discrepancies through discussion and consensus. Once coding is complete, excerpts will be organized into a structured matrix to compare perspectives across stakeholder groups and to identify salient barriers, facilitators, and implementation needs. This analytic strategy is consistent with methods used in the Preparation Phase and follows established approaches in implementation science.

### Implementation framework (dynamic adaptation process–DAP)

At the time of writing, the Preparation Phase has been completed, including qualitative data collection and preliminary adaptation recommendations. The Adaptation Phase is underway, while the Implementation and Evaluation Phases will take place during the randomized pilot trial.

### Preparation phase (year 1)

#### Overview

Multiple data sources guide the adaptation of the IPS model. Using an implementation science framework previously applied by the US team [16, 30], we identified four key domains for adaptation: systemic, organizational, provider, and participant levels. Our goal is to assess contextual factors and organizational readiness for implementing evidence-based practices in Jalisco. We use a framework developed in prior Latin American studies to document implementation barriers and facilitators [31, 32].

#### Data collection

- Systemic level: we conducted six semi-structured interviews with influential policymakers overseeing current mental health policies in Jalisco, focused on potential adjustments to align IPS within the mental health system, state legislation, and labor market. We included The National Employment Service to gather insights on employability for people with disabilities, including mental health conditions. We also explored how policymakers perceive the potential of IPS to meet local needs.
- Organizational level: we interviewed five health managers to examine the conditions required to implement and sustain IPS. Topics included existing resources, anticipated challenges, and strategies to integrate IPS into public services in Jalisco.
- Provider level: two focus groups with mental health providers (6–8 per group) discussed current practices, training needs, and the structure of existing supported employment services. The discussions addressed pragmatic factors (e.g., staffing, funding), organizational culture (e.g., leadership), and coordination with other public sectors.
- Participant level: we held two focus groups of people with SMI and two with family members (6–8 participants each) to explore the perceptions of IPS acceptability and identify ways to adapt the model to meet users’ needs.

## Data analysis

We analyzed the interview and focus group data using a flexible coding approach [33, 34], as applied in prior work by the US team in Latin America [31, 32]. This process included audio recording and professional transcription. The analytic process began with a priori codes informed by the multilevel structure of the interview guides (systemic, organizational, provider, participant), followed by the identification of emergent codes during transcript review. Coding was performed by multiple team members who met regularly to refine the codebook and resolve discrepancies through consensus. We then organized coded excerpts into a matrix aligned with the four implementation levels to facilitate comparison across stakeholder groups and to identify salient barriers and facilitators.

### Adaptation phase

The Research Adaptation Team reviews recommendations from the previous phase and incorporates proposed modifications to the IPS model. These recommendations are based on contextual findings, including how local factors may influence the intervention curriculum and considerations for potential scale-up. The team includes Spanish-speaking trainers from the US, local trainers, and the core research group, with regular participation from health managers, service providers, and study consultants (e.g., policymakers). These stakeholders do not formally adopt the IPS model; rather, they contribute to its adaptation by identifying contextual barriers and facilitators. Their insights help inform implementation strategies that preserve fidelity to core IPS principles while ensuring cultural and systemic relevance in the Jalisco context.

Following methods used in prior Latin American studies that adapted evidence-based practices [31, 32]. Twice a month meetings follow a participatory group discussion format to align the intervention with the Jalisco context and support its operational translation. IPS trainers in New York contribute insights on implementation challenges observed in similar contexts. The team systematically reviews intervention materials and discusses lessons learned from international experiences, in collaboration with IPS Center leaders. They make decisions regarding content, delivery strategies, and objectives to preserve the fidelity of core IPS elements during adaptation. The team identified required modifications to support local implementation, which may include adjustments to the training process, content delivery, language, and modality [15].

### Implementation phase

The Research Adaptation Team, in coordination with the IPS Center, identifies and trains IPS Employment Specialists affiliated with the National Employment Service in Jalisco. These specialists participate in a 4-day, in-person training that introduces the IPS model, followed by structured technical assistance. Support includes individual and group supervision, case consultations, and sessions addressing specific topics requested by participants. Role-specific calls are scheduled biweekly during the first 6 months and then shift to a monthly format, with additional meetings organized as needed.

During the Implementation Phase of the randomized pilot trial, monthly consultation calls focus on team operations. A learning management system provides access to training modules, event registration, module completion tracking, attendance records, and evaluation tools, following the model established by the IPS Center in New York. The Research Adaptation Team and IPS Center representatives continue to deliver training to support local adaptations, adjust instructional content, and address emerging barriers to implementation (e.g., staff shortages, stigma).

To manage challenges, the team uses a structured problem-solving approach informed by prior experience, local knowledge, and relevant literature. External experts participate in specific meetings when issues arise that require additional technical, contextual, or cultural expertise. These experts include IPS trainers from the IPS Center in New York, implementation science specialists, and local consultants with experience in disability rights, labor inclusion, gender and sexual diversity, and youth mental health. Their input is sought in situations where emerging barriers relate to cultural or social factors that can influence engagement in employment, such as stigma related to mental illness, gender roles in the workplace, discrimination based on sexual orientation, or limited economic opportunities for young adults. This tailored consultation process supports context-sensitive problem-solving and helps ensure that IPS implementation remains aligned with both core model principles and local cultural dynamics.

### Evaluation phase

This phase assesses how IPS is implemented in practice and identifies barriers and facilitators that emerge during real-world delivery. Mixed-methods data will be collected at approximately 6 and 12 months from key stakeholders, including participants, IPS Specialists, clinical staff, and administrators. Qualitative interviews will explore experiences with implementation, perceptions of acceptability and feasibility, and contextual factors influencing the adoption of IPS principles. Quantitative indicators, including fidelity scores and service-level metrics, will complement the qualitative findings. All implementation data will be organized across four levels—systemic, organizational, provider, and participant—to allow structured comparison and to capture how challenges and adaptations evolve over time.

### Data collection

- Systemic level: five semi-structured interviews with influential policymakers in Jalisco.
- Organizational level: five semi-structured interviews with health managers in the Jalisco mental health system.
- Provider level: two focus groups with provider staff (both involved and not involved in providing IPS) exploring acceptance or resistance to the model. The discussion follows a funnel structure, starting from general perceptions and narrowing toward concrete, locally relevant examples.
- Participant level: two focal groups with PSMI (*n* = 6–8) and one with family members (*n* = 6–8), exploring perceptions of IPS acceptability and the role of families in recovery-oriented activities.

## Data analysis

As in the Preparation Phase, qualitative data from the Evaluation Phase will be analyzed using a flexible coding approach. Transcripts will first be reviewed using the existing multilevel codebook (systemic, organizational, provider, participant), with additional inductive codes added as needed. Coded excerpts will then be transferred into a structured matrix to compare implementation experiences across stakeholder groups and to identify patterns related to barriers, facilitators, and adaptations. This analytic strategy enables triangulation across data sources and supports the development of a heuristic model to inform future IPS implementation in Mexico.

### Monitoring

#### Data monitoring and reporting

The coordinating center, composed of the Principal Investigator, study coordinators, research staff, data analyst, and statistician, will hold monthly meetings to oversee daily trial operations. These include participant enrollment, data collection, intervention delivery, and compliance with regulatory requirements. An external steering committee, including experts in psychiatry, psychosocial rehabilitation, biostatistics, and mental health advocacy, will convene quarterly to review study progress and participant safety. This committee is independent of the sponsor and study team, and will provide guidance on trial continuation, protocol modifications, or early termination, based on safety indicators and study performance.

If the National Institute of Mental Health (NIMH) does not assign a Data and Safety Monitoring Board (DSMB), an independent board of local experts in Mexico will assume this role. No formal interim efficacy analyses are planned, as this is a feasibility study. However, the DSMB will review the final Data Safety Monitoring Plan, study protocol, protocol amendments, reportable adverse events, and annual progress reports.

#### Adverse event reporting and harms

The latest version of the REDCap system will be used to document both scheduled assessments and unscheduled (anytime) events, including adverse events. If a participant shows signs of psychological or emotional distress, the assigned psychiatrist at the Center for Comprehensive Care in Long-Stay Mental Health will be notified by the research team. When necessary, an immediate and supervised referral will be arranged to address concerns about potential self-harm. Research staff will be trained to recognize indicators that require urgent clinical attention, such as agitation, aggression, tearfulness, fear, anger, suicidal ideation, or worsening psychiatric symptoms. All adverse events and actions taken will be documented and reviewed periodically by the DSMB.

#### Frequency and plans for auditing trial conduct.

The study coordinator will conduct monthly quality assurance audits focused on informed consent procedures, eligibility verification, intervention delivery according to fidelity standards, and accuracy of data collection and entry. In addition, an independent auditor not affiliated with the coordinating center will conduct semi-annual reviews of overall trial conduct. These audits will follow Good Clinical Practice (GCP) guidelines and include verification of source documents and evaluation of compliance with regulatory policies.

## Ethics and dissemination

### Ethics approval and consent to participate

National Institute of Psychiatry Research Ethics Committee for human beings CONBIOETICA. −09-CEI-010–20170316 approved on October 17th, 2022 the protocol number CEI/C/055/2022 and valid for 3 years from October 17th, 2022 to October 17th, 2025. At the time of manuscript preparation, this approval remains valid. As required for studies with extended follow-up, the research team will submit all continuing review and renewal documentation to ensure uninterrupted ethical approval throughout the duration of the trial. All participants will provide written informed consent prior to enrollment.

### Protocol amendments

Any substantive changes to the study protocol will be submitted as formal amendments to the reviewing Institutional Review Board (IRB) prior to implementation. Required updates will also be posted to ClinicalTrials.gov in accordance with registry guidelines. When protocol modifications directly affect participants, study investigators will meet with those affected to explain the rationale, obtain renewed informed consent, and address any questions. Broader amendments that do not directly impact participants will be summarized during the annual continuing review process to the oversight IRB, along with aggregate data on participant retention and adherence.

### Informed consent process

After presenting the IPS pilot study to clinical staff at the Center for Comprehensive Care in Long-Stay Mental Health, clinicians will identify potential participants and refer them to the psychosocial rehabilitation service for an initial informational session. During this session, a trained research assistant will assess eligibility and explain the study’s purpose, procedures, potential risks, and benefits. The assistant will also assess the individual’s capacity to consent using the “Participant Comprehension Assessment Form for Consent.” If the individual lacks capacity to consent, no surrogate consent will be sought. Instead, they will be referred to standard care services.

Individuals who meet all inclusion criteria will be invited to a second informational session, during which the research assistant will review the informed consent form in detail. Participants will be informed that participation is voluntary and that they may withdraw from the study at any time. A copy of the informed consent form will be provided and explained. If the individual agrees to participate, they will sign the form; signed copies will be stored in the research file and provided to the participant. Participants will also be asked for permission to contact a relative. If granted, the relative will receive an explanation of the study’s goals, procedures, risks, and benefits. Other respondents—such as stakeholders at the system, organizational, and provider levels—who are involved in qualitative interviews or focus groups will undergo a tailored consent process, given the potential for disclosure of sensitive or identifiable information.

### Confidentiality

Participant confidentiality will be maintained through a series of safeguards designed to minimize the risk of identification and unauthorized data access. No personally identifiable information, such as names, contact details, or medical record numbers, will be entered into the research database. Each participant will be assigned a randomly generated study identification number.

A linkage file containing identifying information and corresponding study IDs will be stored separately in a locked cabinet at the Center for Comprehensive Care in Long-Stay Mental Health. Only authorized research personnel and clinicians will have access to this file. The study database itself will remain fully de-identified, and no electronic links between identifiers and study data will be created.

To enhance data security, the study will use a tiered dataset structure involving three files. These files will be stored in separate secure locations and require combined access to re-identify a participant. This method, previously approved by Institutional Review Boards (IRBs), has been used successfully in prior studies involving psychosocial interventions.

Data will be stored on password-protected computers with locked screen savers. Internet data transmission will be encrypted and restricted to de-identified information. No identifiable data will be transferred to Columbia University or the New York State Psychiatric Institute. All study data will remain in Mexico. The research data center employs a firewall and intrusion detection system to monitor network activity. All research staff are trained in human subjects protection and confidentiality protocols and will receive periodic reinforcement to ensure ongoing compliance.

### Declaration of interests

The authors declare that they have no competing interests.

### Access to data

Personally identifiable information, such as contact details collected on original forms, will be accessible only to the Principal Investigator and designated research staff. These records will be stored in a locked office at the Center for Comprehensive Care in Long-Stay Mental Health in Jalisco. No identifiable data will be shared outside the local research team.

### Ancillary and post-trial care

This study qualifies as minimal risk, as the probability and magnitude of harm or discomfort are no greater than those typically encountered in daily life or during routine physical or psychological evaluations. Nonetheless, researchers will take all appropriate measures to safeguard participant confidentiality.

Potential risks include emotional discomfort or anxiety associated with being identified as a person with SMI, or distress triggered by sensitive interview questions. Some participants may find the assessments repetitive or emotionally taxing, particularly if they recall difficult memories or if their expectations regarding employment outcomes are unmet. Participants will retain access to their usual clinical care throughout the study and will be referred to emergency services if needed at any time.

### Dissemination plans

The research team is committed to ensuring broad dissemination of study findings in alignment with NIH data sharing policies. A de-identified dataset and the corresponding statistical analysis code will be made publicly available through the INPRFM institutional repository within 3 years of the primary publication.

This approach aims to promote transparency, enable secondary data analyses, and support replication efforts to evaluate the effectiveness of the IPS model across different populations and contexts. Study results will also be shared through peer-reviewed journal articles, academic conferences, and stakeholder engagement meetings. These efforts are intended to inform mental health policy, service delivery, and future implementation strategies.

## Discussion

This study protocol describes a phased, mixed-methods pilot randomized controlled trial designed to evaluate the feasibility and contextual fit of the IPS model for people with SMI in a Latin American public mental health system. The collaboration between the Jalisco Institute of Mental Health, the National Institute of Psychiatry in Mexico, Columbia University, and the Dartmouth Psychiatric Research Center brings together local clinical expertise and international experience in supported employment and implementation science. By applying the Dynamic Adaptation Process (DAP), the study seeks to integrate IPS into routine services through an iterative sequence of preparation, adaptation, implementation, and evaluation.

The mixed-methods design will generate complementary quantitative and qualitative evidence on feasibility outcomes, including recruitment and retention, IPS fidelity, and stakeholder acceptability. Quantitative analyses will provide preliminary estimates of employment, financial, and health-related outcomes, while qualitative data will illuminate contextual barriers and facilitators at systemic, organizational, provider, and participant levels. Together, these findings are expected to inform refinements to the adapted IPS model, the training and supervision strategy, and the operational procedures required for a future definitive trial.

Several challenges are anticipated. Recruitment and retention may be difficult given the clinical complexity and potential instability of the target population, as well as structural barriers to consistent engagement in services. To address this, the protocol incorporates assertive outreach strategies, detailed locator information, and financial incentives for participation, building on approaches that have yielded high follow-up rates in previous IPS studies. Variability in the mode of intervention delivery (e.g., in-person versus virtual contacts) may also introduce heterogeneity in exposure to IPS services. While such flexibility is intended to enhance accessibility, all deviations from standard delivery will be documented and considered in interpreting feasibility and outcome data.

An additional limitation is that the specific thresholds used within the traffic-light progression framework were not empirically calibrated for this setting prior to the pilot trial. Although the criteria were defined a priori and informed by methodological guidance for external pilot studies, feasibility benchmarks often require refinement once empirical data from the pilot become available. In line with current recommendations, progression criteria in pilot trials should be interpreted as decision-support guidelines rather than rigid statistical rules and may be refined using the evidence generated during the pilot phase. Therefore, the thresholds proposed in this study will be interpreted alongside qualitative findings, implementation context, and fidelity assessments when determining readiness for a future definitive trial [35].

Maintaining fidelity to the IPS model is another key challenge in low- and middle-income settings, where staffing patterns, service availability, and labor markets differ from those in high-income countries. The study addresses this by incorporating intensive initial training, ongoing supervision from experienced IPS trainers, structured fidelity assessments, and regular feedback loops between local teams and the IPS Center. These strategies aim to minimize protocol drift while allowing contextually appropriate adaptations.

Although sustainment and scale-up are central topics in the broader IPS and implementation science literature, they are not formal outcomes of this pilot study. Our focus is on early-stage implementation processes and feasibility parameters that will inform later decisions regarding broader system integration. Similarly, adoption is not measured as a primary outcome; instead, we document contextual factors and early indicators that may influence subsequent uptake of IPS within the Jalisco mental health system [36].

Despite these challenges, the pilot trial is expected to make a substantial contribution to the IPS and global mental health literature. It will provide one of the first systematic evaluations of IPS implementation in Mexico, generate empirical data on feasibility and preliminary outcomes in a public-sector setting, and offer detailed information on the adaptation process in a middle-income country. The lessons learned regarding training, fidelity monitoring, and multilevel contextual barriers may support future efforts to implement evidence-based supported employment in other regions of Latin America and similar resource-constrained settings.

### Strategies to overcome potential study limitations

To address the potential for sample attrition, the study employs strategies such as assertive outreach, frequent contacts, and the collection of detailed tracking information at each interview. These measures, combined with financial incentives for participation, are designed to maintain high retention rates throughout the study.

Maintaining treatment fidelity is critical to the validity of the study. The implementation phase allows for the thorough training of IPS staff and the development of a fidelity monitoring system. This system includes regular reviews and adjustments to ensure adherence to the IPS model, minimizing the risk of protocol drift.

Recruitment and retention may represent key challenges given the clinical complexity and potential instability of the target population. To mitigate these risks, the study includes assertive outreach, frequent contact with participants, and the collection of detailed tracking information during each assessment point. Financial incentives will also be provided to support retention and minimize attrition.

Variability in the mode of intervention delivery (e.g., in-person or virtual sessions) may introduce heterogeneity in participant experiences and intervention exposure. While intended to increase accessibility, this variability may affect implementation consistency and complicate the interpretation of outcomes. All deviations from the standard delivery format will be systematically documented and considered in the analysis.

Ensuring fidelity to the IPS model is another essential component for study validity. The implementation phase allows for comprehensive training of IPS staff and the establishment of a fidelity monitoring system. This system includes routine supervision, structured fidelity assessments, and mechanisms for ongoing feedback and corrective action to prevent protocol drift over time.

### Trial status

The trial has not yet commenced participant recruitment. The study has received approval from the INPRFM Ethics Committee (approval number CONBIOÉTICA-09-CEI-010–20170316) and the INPRFM research committee. The study is registered with ClinicalTrials.gov (NCT06019247) and updates will be provided annually.

## Data Availability

No datasets were generated or analyzed during the preparation of this study protocol. Data generated during the course of the study will be available from the corresponding author upon reasonable request, subject to ethical approval and institutional regulations governing the sharing of clinical research data.

## Abbreviations

IPS: Individual Placement and Support
Eg: Example
SMI: Serious mental illness
DSMB: Data and Safety Monitoring Board
INPRF: National Psychiatry Institute “Ramón de la Fuente Muñiz
